# Extended Adjuvant Endocrine Treatment in Luminal Breast Cancers in the Era of Genomic Tests

**DOI:** 10.3390/ijms232113604

**Published:** 2022-11-06

**Authors:** Mariarosaria Saponaro, Luigi Annunziata, Antonella Turla, Ilaria Viganò, Michele De Laurentiis, Mario Giuliano, Lucia Del Mastro, Filippo Montemurro, Fabio Puglisi, Carmine De Angelis, Giuseppe Buono, Francesco Schettini, Grazia Arpino

**Affiliations:** 1 Department of Clinical Medicine and Surgery, University of Naples Federico II, 80100 Naples, Italy; 2 Department of Medical and Surgical Specialties, Radiological Sciences and Public Health, University of Brescia, Medical Oncology, ASST Spedali Civili, 25100 Brescia, Italy; 3 Medical Oncology, Ospedale Valduce, 22100 Como, Italy; 4 Department of Breast and Thoracic Oncology, National Cancer Institute, Fondazione G. Pascale, 80100 Naples, Italy; 5 Interdepartmental Center of Clinical and Translational Sciences (CIRCET), Federico II University, 80100 Naples, Italy; 6 Department of Medical Oncology, IRCCS Ospedale Policlinico San Martino, 16100 Genova, Italy; 7 Breast Unit, Istituto di Candiolo, FPO-IRCCS, 10127 Candiolo, Italy; 8 CRO Aviano, National Cancer Institute, IRCCS, 33081 Aviano, Italy; 9 Department of Medicine, University of Udine, 33100 Udine, Italy; 10 Medical Oncology Department, IDIBAPS, Hospital Clinic of Barcelona, 08000 Barcelona, Spain; 11 Faculty of Medicine and Health Sciences, University of Barcelona, 08000 Barcelona, Spain

**Keywords:** late recurrence, hormone receptor-positive breast cancer, extended endocrine therapy, clinical score, genomic assays

## Abstract

In patients with early-stage endocrine receptor-positive (ER+) breast cancer (BC), adjuvant endocrine therapy (ET) for 5 years is the standard of care. However, for some patients, the risk of recurrence remain high for up to 15 years after diagnosis and extended ET beyond 5 years may be a reasonable option. Nevertheless, this strategy significantly increases the occurrence of side effects. Here we summarize the available evidence from randomized clinical trials on the efficacy and safety profile of extended ET and discuss available clinical and genomic tools helpful to select eligible patients in daily clinical practice.

## 1. Introduction

About 70% of breast cancers (BC) are endocrine receptor-positive (ER+), and the cornerstone of treatment is endocrine therapy (ET) [1,2]. Adjuvant ET reduces BC recurrence and improves overall survival (OS) in ER+ patients [3,4]. Until the early 2000s, 5 years of ET with tamoxifen was the treatment of choice in both premenopausal and postmenopausal women. The past few decades have seen the development of other treatment regimens, among which aromatase inhibitors (AIs) and a combination of these with tamoxifen. In pivotal randomized trials of postmenopausal patients, AIs (either letrozole, anastrozole. or exemestane) given upfront for 5 years (instead of tamoxifen) or given for 2–3 years after 3–2 years of tamoxifen are better than tamoxifen alone in terms of both disease-free survival (DFS) and OS, thus becoming the standard adjuvant therapy in postmenopausal women [5,6,7]. However, ER+ BC has a long natural history. Indeed, some women remain at risk of recurrence for at least 15 years after diagnosis [8,9]. The benefit of the extended use (beyond 5 years) of adjuvant ET in postmenopausal women, has been the subject of several trials. Two of the early trials, namely the ATLAS and aTTOm trials, demonstrated that extending the use of adjuvant tamoxifen from 5 to 10 years in pre- and postmenopausal women resulted in an additional reduction in the risk of BC recurrence and mortality [10,11]. Consistently, other trials have shown that adding an AI for 5 years after initial tamoxifen for 2–3 years is also beneficial in reducing BC recurrence [12,13,14,15] and that continuing an AI for a total of 7–10 years after the initial 5 may also play a role in further recurrence risk reduction [16,17,18,19,20,21]. However, the benefit of such an approach remains controversial and current clinical practice guidelines state that an individualized approach on the basis of residual risk reduction and tolerability is needed [22]. Furthermore, ET can be escalated with the addition of 5 years of ovarian function suppression with either tamoxifen [23] or an AI in the premenopausal setting [24]. In this scenario, prognostic and predictive tools have been developed to assist clinicians and patients in adjuvant treatment decisions.

In this review, we will analyze published data about the efficacy and tolerability of extended adjuvant treatment in both the pre- and postmenopausal settings and will also discuss the clinical validity and utility of the currently available genomic tools to select patients for this strategy in clinical practice.

## 2. Results

### 2.1. Efficacy and Safety of the Extended Tamoxifen Therapy

The aTTom trial [10] enrolled about 7000 patients who had completed 5 years of adjuvant tamoxifen treatment. They were randomized to continue tamoxifen for an additional 5 years or discontinue it permanently. The extended strategy resulted in a 3% reduction in the risk of late recurrence (16.7% vs. 19.28%, *p* = 0.003) and in a 1.4% reduction in the risk of BC mortality (11.3% in the extended group vs. 12.7% in the control group). Prolongation of tamoxifen slightly increased the incidence of endometrial cancer (2.94% in the extended group vs. 1.29% in the control group). However, most of the endometrial cancers were low-grade and curable by surgery.

Similar to the aTTom trial [10], the ATLAS trial [11] included about 13,000 patients that were randomized to discontinue adjuvant tamoxifen treatment after 5 years or to continue treatment for a further 5 years. Only half of the patient population had an ascertained ER+ BCs. The risk of recurrence at 5–14 years for patients with ER+ BC was 21.4% in the extended group vs. 25.1% in the control group (*p* = 0.002), meaning an additional 3.7% reduction in the absolute risk of recurrence with extended ET. Similarly, BC mortality at 5–14 years was 12.2% in the extended group vs. 15% in the control group (*p* = 0.01), meaning an additional 2.8% reduction in the absolute risk of death for the extended ET compared to the control. At the same time, in the ATLAS trial, a prolongation of tamoxifen therapy for 10 years was associated with an increased risk of endometrial cancer (3.1% vs. 1.6% in the extended vs. control arms, respectively, *p* = 0.0002), as well as an increased thromboembolic risk, mostly driven by pulmonary embolism (relative risk (RR): 1.87, 95% confidence interval (CI): 1.13–3.07, *p* = 0.01) compared to standard treatment duration.

### 2.2. Efficacy and Safety of Extended Therapy with AIs

The GIM4 [12] trial enrolled 2056 postmenopausal patients without evidence of disease after 2–3 years of adjuvant tamoxifen. Patients were randomized to receive letrozole for 3–2 years (control group, standard overall duration) vs. 5 years (extended group). After a follow-up of about 12 years, extended letrozole resulted in a significant improvement in DFS (HR: 0.78, 95% CI: 0.6–0.93, *p* = 0.006) and OS (HR: 0.77, 95% CI: 0.60–0.98, *p* = 0.036). The subgroup analysis showed that extended letrozole was more likely to improve DFS in patients with tumors expressing both estrogen and progesterone receptors, which were HER2-positive and N-. The greater benefit in the N- disease could be due to the enrollment of patients disease-free after 2–3 years of tamoxifen, thus with a better prognosis. The main AEs were arthralgia, myalgia, and osteoporosis. The incidence of osteoporosis was increased in the extended group (8.3% vs. 4.7%). No differences were observed for hypercholesterolemia, cardiovascular diseases, or bone fractures.

In the MA.17 [13] trial, 5187 postmenopausal patients who had completed adjuvant treatment with tamoxifen for 5 years were randomized to continue treatment for an additional 5 years with letrozole vs. placebo. At a 30-month follow-up, the patients on extended treatment with letrozole had a longer DFS than the non-treated group (4-year DFS 93% vs. 87%, HR: 0.57, 95% CI: 0.43–0.75, *p* < 0.0001), while OS did not differ between the two groups. Letrozole was equally effective in patients with N+ and node-negative (N-) disease. Regarding adverse events (AEs), a higher incidence of arthro-myalgia and osteoporosis was observed in the letrozole group than in the placebo group (3.6% vs. 2.9% *p* = 0.07). No significant difference was found in the incidence of cardiovascular disease and in new bone fractures between the two groups. The study was stopped prematurely following an interim analysis showing the superiority of the extended arm in terms of DFS and patients randomized in the placebo arm were allowed to switch to the letrozole group. A subsequent analysis, at a follow-up of 64 months adjusted for crossover from placebo to active treatment showed that extended treatment with letrozole was significantly superior to placebo in terms of DFS (HR: 0.58, 95% CI: 0.47–0.72, *p* < 0.001), distant DFS (HR: 0.68, 95% CI: 0.52–0.88, *p* = 0.004), and OS (HR: 0.77, 95% CI: 0.60–0.96, *p *= 0**.02) [14].

The DATA [15] trial enrolled about 1900 patients and compared extended treatment with anastrozole for 6 years vs. anastrozole for 3 years after 2–3 years of tamoxifen therapy. The results showed no significant benefit in prolonging ET up to a total of 8–9 years, with a disease-free survival (DFS) of 83.1% in the extended group vs. 79.4% in the control group (hazard ratio (HR): 0.79, 95% CI: 0.62–1.02, *p* = 0.066). Moreover, no difference was observed between the two groups in terms of OS. A post hoc subgroup analysis showed that treatment with anastrozole for 6 years improved DFS in patients who were both estrogen and progesterone receptor-positive, as well as axillary lymph node-positive (N+) and larger primary tumor size (≥T2). In the extended group, compared to the control, there was a significant increase in arthralgias (58% vs. 53%) and osteopenia or osteoporosis (21% vs. 16%). There were no increases in cardiovascular risk or other side effects.

At the 10-year follow-up, the final results confirmed the previous evidence. In fact, the 10-years adapted disease-free survival (aDFS) was 69% in the extended group vs. 66% in the control group (HR 0.86, 95% CI: 0.72–1.01, *p* = 0.0.73). However, extended treatment with letrozole for 6 years improved the aDFS in patients who were both estrogen and progesterone receptor positive and had a N+ disease (HR 0.74, 95% CI: 0.59–0.93, *p* = 0.011). No difference was observed between the two groups in terms of OS [25].

In the IDEAL study [16], 1824 postmenopausal patients already treated with adjuvant ET for 5 years (tamoxifen alone, tamoxifen +AI, AI alone) were randomized to receive letrozole for a further 2.5 vs. 5 years. Neither DFS (*p* = 0.49) nor OS (*p* = 0.79) differed between the two groups. However, the risk of a second primary BC was lower in the extended group (HR: 0.39, 95% CI: 0.19–0.81 *p* = 0.01). Subgroup analysis did not reveal a category of patients that benefitted more than the others from extended ET. The most frequent AEs were arthralgia (13.2% in the 2.5 years group vs. 14.75 in the 5 years group), hot flashes (10.5% vs. 13.1%), osteoporosis (7.5% vs. 12.7%), and fatigue (7.5% vs. 9.7%). A total of 368 patients discontinued therapy because of AEs (17.3% in the 2.5 years arm vs. 23.5% in the 5-year arm).

The NSABP-B42 [17,18] trial enrolled 3966 postmenopausal patients who had received 5 years of adjuvant ET (either tamoxifen for 2–3 years followed by an AI for 3–2 years or upfront AI). Patients were randomized to receive letrozole or placebo for an additional 5 years. The first results, after 6.9 years of follow-up, showed no difference between the two groups in either DFS (*p* = 0.048) or in OS (*p* = 0.22). Subgroup analyses were non-significant, although they pointed towards a slightly better performance for extended letrozole in patients who had received mastectomy or previous tamoxifen. The most common AEs were arthralgia and back pain. However, data analysis after 9.3 years of follow-up showed that extended letrozole improved DFS (HR: 0.84, 95% CI: 0.74–0.96, *p* = 0.011), BC free-interval (HR: 0.74, 95% CI: 0.61–0.91, *p* = 0.003), and distant recurrence-free intervals (HR: 0.71, 95% CI: 0.55–0.93, *p* = 0.01). Overall survival did not differ between the two groups. Prolongation of treatment to 10 years did not cause a significant increase in the incidence of AEs compared to previous analyses.

The MA.17R [19] trial enrolled approximately 1900 postmenopausal patients who had completed 5 years of adjuvant ET (aromatase inhibitor with or without tamoxifen). Patients were randomized to receive letrozole or placebo for another 5 years. Extended letrozole significantly improved DFS (HR: 0.66, 95% CI: 0.48–0.91, *p* = 0.01), although no difference in OS was observed between the two groups. Letrozole proved to be superior to placebo, independently of tumor size, axillary nodal status, prior chemotherapy, or previous ET. In addition, extended letrozole further reduced the risk of contralateral BC (HR: 0.42, 95% CI: 0.22–0.81 *p* = 0.007). The most common AEs were arthralgia, hot flushes, and fatigue in both groups. However, newly diagnosed osteoporosis, which entailed an increased (11% vs. 6%) risk of bone fractures, was higher in the letrozole group (11% vs. 6%).

The SOLE study [20] enrolled 4884 postmenopausal patients with ER+, lymph node-positive, operable BC who had completed 4–6 years of adjuvant ET (either tamoxifen for 2–3 years followed by an AI for 3–2 years or upfront AI) and who were randomized to either extended intermittent letrozole (9 months followed by 3 months of discontinuation for the first 4 years, continuously for the last year) or continuous extended letrozole for 5 additional years. Concurrently, the SOLE-EST sub-study enrolled 104 patients in the SOLE trial to monitor their circulating estrogen levels. The aim was to evaluate whether intermittent letrozole led to an increase in plasma estrogen levels, thereby prolonging the sensitivity of cancer cells to AIs, as previously observed in animal models. The results of the SOLE study showed no difference in either DFS (*p* = 0.64) or OS (*p* = 0.19) between the two groups. The subgroup analyses, based on age, BMI, prior ET, tumor size, tumor grade, number of positive nodes and HER2 status, did not reveal any significant differences. Similarly, no difference was observed in AEs, although a better quality of life was reported in the intermittent arm. Data from the SOLE-EST study showed that despite an increase in circulating estrogen levels during the letrozole withdrawal period, the latter modification was not significantly associated with the clinical effects of treatment measured by quality-of-life scales or grip strength. Although no correlation with survival was reported, the overall DFS and OS suggest that extended adjuvant ET with an intermittent schedule could safely be administered when required.

The ABCSG-16 [21] study enrolled 3484 postmenopausal patients who had completed 5 years of adjuvant ET with tamoxifen, Ais, or both sequentially. Patients were randomized to continue treatment with anastrozole for 2 vs. 5 years. The study showed no advantage in 10-year vs. 7-year treatment in either DFS (*p* = 0.9) or in OS (HR: 1.02, 95% CI: 0.83–1.25). Subgroup analyses did not show significant differences in terms of specific subgroups. The most common adverse event was osteoarthritis (4.3% in the 5-year group vs. 1.7% in the 2-year group). On the contrary, a greater risk of bone fractures was reported in the 5-year than in the 2-year group (6.3% vs. 4.7%).

Extended ET trial results are summarized in Table 1.

### 2.3. Meta-Analyses of Trials on Extended ET

Gray et al. [26] reported the data of 11 trials of extended adjuvant ET plus the data of >24,000 patients during the San Antonio Breast Cancer Symposium 2018. The extended therapy beyond 5 years resulted in an overall 35% reduction in the risk of relapse for patients treated with tamoxifen. In patients undergoing treatment with AIs, with or without tamoxifen switch, the risk of relapse was reduced by 20%. The risk reduction seemed to be prevalent in the first 2 years after tamoxifen, but only modest after previous AIs. This suggests that the optimal duration for extended therapy could be limited to 7–8 years. Regarding AEs, extended therapy resulted in a 25% increase in the risk of bone fractures.

In a more recent meta-analysis, Chen et al. [27] found that prolonging endocrine treatment beyond 5 years can improve DFS (HR 0.77 CI 0.68–0.89, I^2^ 66.4%, random effects model). Although this was evident only in particular categories of patients, namely those with ER+ tumors, a primary tumor size >2 cm, previous ET with tamoxifen or tamoxifen followed by AIs. Conversely, no advantage was found in patients who did not meet these criteria. The analysis confirmed an increased risk of osteopenia, osteoporosis, and bone fracture with extended AI therapy. Lastly, no substantial differences were observed between a total ET duration of 7–8 years vs. 10 years.

### 2.4. Clinicopathological and Genomic Biomarkers for Risk Stratification and Patient Selection

Considering the variable clinical benefit deriving from extended ET and the increased toxicity rate, optimal patient selection is crucial. In this perspective, clinicopathological criteria and prognostic genomic assays might be of help. In a recent expert consensus, Garutti et al. [28] examined various clinical pathological factors to identify the clinical recurrence risk factors. The most important risk factors in defining a poor prognosis in early BC were high histological grade, histological type, i.e., pure tubular, pure mucinous, and pure cribriform histologies are at lower risk, N+ disease, tumor size T2 or greater, high level of Ki67 expression, low ER/PgR expression, residual cancer burden after neoadjuvant treatment and high-risk class at the genomic signature.

#### 2.4.1. Clinical Treatment Score at 5 Years (CTS5)

The CTS5 is a clinical score that estimates the risk of late recurrence in women with ER+ early BC after standard 5-year adjuvant ET. The score was developed by Dowsett et al. [29] based on data from the ATAC trial and validated in the BIG1-98 trial of adjuvant letrozole and/or tamoxifen alone or sequentially. The score is based on several clinicopathological parameters, namely age, tumor size, quadratic tumor size, nodal status, and tumor grade, and is calculated according to the following formula: 0.471 × nodes + 0.980 × (0.164 × size − 0.003 × size 2 + 0.312 × grade + 0.03 × age). The score classifies patients into three risk categories: low risk (<5%), intermediate (5–10%), and high risk (>10%) of distant recurrence after 5 years of ET.

Tajiri et al. [30] investigated the clinical usefulness of CTS5 in a study of 560 postmenopausal patients with ER+ BC. After 5 years of adjuvant ET, patients were divided into three risk categories based on CTS5. The incidence rate of distant relapses was higher in patients in the intermediate-high risk group than in patients in the low-risk group. The incidence rate at 5–10 years was 1.5% in the low-risk group, 3.8% in the intermediate risk group, and 7% in the high-risk group. The incidence rate at >10 years was 0.5% in the low-risk group, 2.8% in the intermediate risk group, and 4.2% in the high-risk group. The DFRS in the intermediate (HR 4.33 95% CI 1.20–20.1 *p* = 0.0246) and high risk (HR 6.48 95% CI 1.87–29.7 *p* = 0.0030) group was lower than in the low-risk group. These data suggest that patients at CTS5-intermediate/high risk of recurrence might be good candidates for extended ET.

Richman et al. [31] conducted another retrospective study on 2428 women with ER+ early BC. At a median follow-up of 13.4 years, the 10-year distant relapse risk was 2.9% for the CTS5-low group, 7.2% for the CTS5-intermediate group and 12.9% for the CTS5-high risk group. No significant interaction was observed between chemotherapy and CTS5. These data also suggest that women with a low CTS5 risk are unlikely to benefit from the extension of ET beyond 5 years. Conversely, women with a higher risk might prolong ET.

Finally, CTS5 was shown to be prognostic in the TAILORx trial [32]. In fact, the HR at 5–9 years post-surgery was 1.57 (1.36–1.82, *p* < 0.001) in the overall population (7353 patients). At the subgroup analyses, CTS5 was not prognostic in the arm with risk score (RS) 0–10/ET (HR: 1.34, 95% CI: 0.87–2.07, *p* = 0.19), but predicted late distance recurrences in the arm with RS 11–25/ET (HR: 1.50, 95% CI: 1.17–1.93, *p* = 0.002), RS 11–25/CET (chemo-endocrine therapy) (HR 1.56 95% CI: 1.22–1.98, *p* = 0.0003) and RS 26–100/CET (HR: 1.90 95% CI: 1.23–2.92, *p* = 0.004). Interestingly, age impacted on the prognostic role of CTS5. In fact, the CTS5 was strongly prognostic for late distant recurrence (DR) in patients >50 years (HR: 1.78 95% CI: 1.48–2.14, *p* < 0.0001), while it was less prognostic for women aged 50 years or younger (HR: 1.35 95% CI: 1.01–1.82, *p* = 0.046).

#### 2.4.2. Oncotype DX

Oncotype DX^®^ is a genomic assay that analyzes the expression of 21 genes at the level of fresh-frozen paraffin-embedded breast tumor tissue using RT-PCR technology. It generates a recurrence score (RS) that is able to stratify patients with ER+, N- and N+ (1–3 lymph nodes) early-stage BC by the risk of relapse (low, intermediate, high). Based on this score, it is possible to estimate the potential benefit deriving from the administration of adjuvant chemotherapy, in addition to ET [33,34,35,36,37].

Sixteen of the 21 genes of the Oncotype assay have prognostic value and are related to key biological and molecular characteristics of BC, namely estrogen-dependent signaling and tumor proliferation, while the other five are control genes [38]. Thirteen genes are grouped into four modules, i.e., proliferation genes, estrogenic pathway-related genes, HER2, and invasion mechanisms, that are then weighted in order to obtain an RS, ranging from 0 to 100 [37]. The RS was retrospectively validated by analyzing tumor samples from the prospective randomized controlled adjuvant trials NSABP B-20, ATAC, and SWOG 8814. Cut-off values of <18, 18 ≤ RS, ≤ 31, and >31 were established for low, intermediate and high risk, respectively. However, given the results of the more recent TAILORx prospective randomized study in N- disease, the reference cut-off values were changed to <11, 11–26, and >26 for low, intermediate, and high risk, respectively. Importantly, RS values >26 indicated a greater benefit from adding chemotherapy to adjuvant ET alone in N- disease. Patients <50 years benefitted from adjuvant chemotherapy also in the case of intermediate RS, mostly in the score range 16–25 (6.5% absolute benefit) [37]. The RxPONDER prospective trial of N+ (1–3 lymph nodes) disease also showed that postmenopausal patients at intermediate/low risk of relapse according to RS (<26) were likely to not gain significant benefit from adjuvant chemotherapy, while postmenopausal high-risk and premenopausal patients in all risk categories, always seemed to derive benefit from chemotherapy. For the latter group, the benefit was lower in case of RS < 11 [39]. 

The TransATAC study evaluated the prognostic value of the Oncotype DX RS beyond the first 5 years of ET [40]. The aim of this retrospective trial was to identify molecular characteristics driving different BC gene expression assays and to study their different prognostic significance from samples deriving from the tamoxifen and anastrozole arms of the ATAC randomized trial of adjuvant tamoxifen and anastrozole, alone or in combination [33,40]. The estrogen-related gene module was primarily associated to prognosis within the Oncotype assay, with only moderate prognostic value for the proliferation module. The prognosis item was no longer significant after the first 5 years [40]. 

Accordingly, it was not possible to determine the optimal duration of adjuvant ET based on RS.

#### 2.4.3. Prosigna

The standardized commercially available PAM50 gene expression assay (Prosigna^®^) analyzes the expression of 50 genes associated with estrogen-related signaling, the HER2 amplicon, basal features, and proliferation, as well as five control genes. It is able to assess the intrinsic subtype (IS) of the breast neoplasms analyzed (i.e., Luminal A, Luminal B, HER2-enriched, and basal-like). It can also calculate a score indicative of the risk of recurrence for postmenopausal women with ER+ BC by integrating and weighting the genomic information with primary tumor size and nodal status [41,42,43,44,45].

In the ATAC study, Prosigna was validated to predict the 10-year risk of relapse [44]. In a combined analysis between the ATAC and the ABCSG-8 trials, Prosigna also gave information about the risk of a late relapse (>5 years) in N- BC patients [46,47]. However, the test is not specifically intended to assess the benefit of extended ET. Thus, caution should be exercised when therapeutic assumptions are based on currently available data. In addition, IS provides independent prognostic and predictive information regarding standard clinicopathological features, and when available, they could be used as a tool to assess the endocrine sensitivity of a patient’s tumor. For example, Luminal A and B ER+ tumors are the subtypes that most rely on the estrogen signaling pathway for survival and growth, thus being the most endocrine sensitive [42,48]. Conversely, HER2-enriched and basal-like tumors are less endocrine-sensitive subtypes. Indeed, the latter is not at all responsive to ET, as recently demonstrated in the advanced setting [49,50]. In any case, neither ROR nor IS have been prospectively validated to select patients for extended adjuvant ET strategies.

#### 2.4.4. Mammaprint

Mammaprint^®^ was the first genomic test to be approved by the US Food and Drug Administration and to be validated in a randomized clinical trial, i.e., the MINDACT trial [51], that included triple-negative and HER2-positive disease, as well as N+ cases (1–3 axillary nodes). The test consists of the analysis of the expression of 70 genes and is aimed at defining the risk of developing distant metastases in early-stage BC [51]. In the MINDACT trial, within the cohort of clinically high-risk patients, about 46% of patients who would be candidates for chemotherapy did not receive it because of a Mammaprint low-intermediate risk of relapse. These patients had a 5-year metastases-free rate similar to that of patients undergoing chemotherapy [51]. However, as yet, there are no solid data regarding the potential role of the Mammaprint assay in providing information about prognosis and the possible continuation of adjuvant hormone therapy beyond 5 years.

#### 2.4.5. Endopredict

The Endopredict^®^ (EP) molecular test is based on the RT-PCR analysis of the tumor tissues of the expression of 12 genes, 8 of which are associated with prognosis and 4 reference genes; it ultimately provides a risk score from 0 to 15. The cut-off value of 5 distinguishes between patients at low risk from the ones at high risk of BC relapse. The combination of the molecular score, tumor size, and lymph node status resulted in the clinically applicable version of the EP score (Epclin), with a cut-off set at 3.3 to distinguish between low and high-risk patients [37]. Samples from 1702 postmenopausal patients with ER+/HER2-negative early-stage BC treated with 5 years of ET from the two phase-III adjuvant studies ABCSG6 and ABCSG8 were collected to investigate the ability of EP to estimate early (<5 years) and late (>5 years) risk of relapse. Both EP and Epclin were able to efficiently predict the early and late risk of distant relapse, with Epclin being superior to the EP genomic score alone [35]. Importantly, the low-risk group of women identified by the EPclin score showed an absolute risk of distant metastasis of only 1.8% between 5 and 10 years of follow-up [35]. Hence, this score might be useful to identify women for whom a 5-year adjuvant ET might be sufficient, sparing unnecessary side effects.

#### 2.4.6. Breast Cancer Index

The Breast Cancer Index (BCI) is another molecular test that consists of two biomarkers, i.e., the Molecular Grade Index (MGI) that is based on the expression of the *BUB1B, CENPA, NEK2, RACGAP1*, and *RRM* genes that are related to tumor grade and progression, and the H/I, which is a ratio of the levels of expression of another two genes: HOXB13 and IL17B. The H/I was developed in patients with ER+ N- BC and was able to predict prognosis more accurately than the two genomic signatures alone [52,53]. The discriminating cut-off values between low and high risk are <5.0825 and >6.5025. respectively.

Sestak et al. [54] compared various prognostic multi-gene tests, including BCI, Oncotype DX, Prosigna and EP, and the prognostic value of all assays regarding the risk of early relapse (<5 years) was confirmed. Conversely, only BCI, Prosigna, and EP were able to predict the risk of late relapse (5–10 years).

In the TransATTOM study [55], in which the population was composed of patients previously enrolled in the ATTOM trial, BCI by high H/I expression was predictive of response to ET. The index identified a subgroup of ER+, N+ patients who benefitted significantly from prolonged adjuvant tamoxifen for 10 years, compared to 5 (HR 0.35 95% CI 0.15–0.85 *p* = 0.027 while there was no benefit in prolonged therapy in patients with a low H/I from the BCI (HR 1.07 95% CI 0.69–1.65 *p* = 0.77).

Sgroj et al. [56] evaluated the ability of the BCI to predict the risk of relapse, in a retrospective–prospective analysis conducted on the patient population enrolled in the Ma17 trial. In total, 249 patients, 83 with relapse and 166 recurrence-free, were evaluated, and the H /I index was evaluated in all patients. Most patients were over 50 years of age, stage T1/T2 with node-positive disease. In the placebo group, a high H/I was associated with a worse prognosis than a low H/I index (odds ratio [OR]: 2.24, 95% CI: 1.09–4.61, *p* = 0.03), while in the letrozole group, it has no prognostic significance (*p* = 0.72). The patients with high H/I receive benefits with adjuvant extended letrozole (OR: 0.33, 95% CI: 0.15 to 0.73, *p* = 0.006).

Similar results were obtained in the study conducted by Noordhoek et al. [57]. They conducted a prospective–retrospective translational study of 908 patients enrolled in the IDEAL trial, 454 patients in the 2.5-year arm and 454 patients in the 5-year arm. The H/I index was determined in each patient. In patients in the high H/I group, 5 years of letrozole resulted in a statistically significant benefit (HR: 0.42, 95% CI: 0.21–0.84), with a reduction of 9.8% in the absolute risk of recurrence (*p* = 0.011). No significant reduction in the risk of recurrence (*p* = 0.835) with extended ET was observed for patients in the H/I low group.

Lastly, Mamounas et al. [58], using the BCI H/I index, performed time-dependent secondary analyses (≤4, >4 years) to evaluate the possibility of predicting the benefit of extended therapy with letrozole in the NSABP-B42 trial. A total of 2179 patients were analyzed, 40% of whom had an N+ disease. After 4 years, extended letrozole reduced the risk of distant recurrence in the H/I high group (HR: 0.29, 95% CI: 0.12–0.69, *p* = 0.003) but not in the H/I low group (*p* = 0.28). No difference was observed for the benefit of DR between two groups before 4 years.

## 3. Discussion

In recent years many studies have confirmed the prolonged risk of recurrence of ER+ BC. A double peak of disease recurrence has been found 2–3 years and 7–8 years after surgery [8,9]. Over 50% of recurrences were reported after 5 years of adjuvant ET, and a proportion of distant recurrences occurred even 20 years after surgery [31,32]. In this perspective, extended ET could be a strategy with which to reduce the risk of late recurrences. However, based on the results of extended adjuvant ET trials, there is no clear advantage in prolonging ET beyond 5 years in all patients with ER+ early-stage BC. Indeed, the extended treatment increased the incidence of AEs, such as endometrial cancer after extended tamoxifen therapy in premenopausal women, and arthralgia, myalgia, and osteoporosis after AIs. These AEs can compromise treatment compliance, as shown by the adherence to adjuvant tamoxifen and AIs in the range of 41–88% and 52–91%, respectively, and with an alarming discontinuation rate for tamoxifen within 1 year of 15–20% and for AIs within 2 years of 5–25%. These discontinuation rates significantly and negatively affect patients’ survival, as observed in several real-world study cohorts [59,60,61,62,63,64,65,66,67]. Consequently, it is necessary to identify the type of patients who could benefit most from this therapeutic strategy, thereby minimizing the risk of treatment discontinuation because of AEs and maximizing the benefit to patients.

Importantly, pooled analyses of ET trials have shown that especially in patients with both ER+ tumors, primary tumor size > 2 cm, previous ET with tamoxifen or tamoxifen followed by AIs, the increased risk of AEs might be balanced by a more pronounced benefit in DFS and OS. Moreover, results suggest that a 7–8 year overall duration of treatment could be sufficient, without the need to reach a total of 10 years [26]. 

In general, the selection of patients for extended ET may be based on those at a high risk of relapse, namely young women (<50 years) and patients with large tumors, lymph node positivity, aggressive histologic variants, and/or poor prognostic scores on genomic testing [68]. From this perspective, the CTS5 score might be a useful tool with which to identify patients that could benefit from extended therapy (patients at intermediate and high risk).

A number of genomic assays have been developed in the last few years, namely Prosigna, Oncotype DX, EndoPredict, BCI, and Mammaprint. These assays provide valuable prognostic information for patients with ER+/HER2-negative early-stage BC, and are also used to identify candidates for adjuvant chemotherapy, albeit with different levels of evidence [69]. However, only EndoPredict [35,37], Prosigna [41,42,43,44,45,46,47,48,49,50], and BCI [52,53,54,55,56,57,58] are able to provide reliable prognostic information beyond 5 years from primary diagnosis. Furthermore, BCI can predict tamoxifen benefit and Prosigna can identify the molecular BC subtype (the so-called “intrinsic subtype”) that might be helpful, beyond clinicopathological evidence, to identify tumors with a higher or lower endocrine sensitivity. In any case, no prospective trials have been performed to specifically demonstrate the capability of all these assays to efficiently discriminate patients who benefit or not from extended adjuvant ET. 

It is important to notice, however, that adjuvant therapies in luminal BC are rapidly evolving as cyclin-dependent kinase 4 and 6 (CDK 4/6) inhibitors and PARP inhibitors are now indicated in this setting. 

In detail, for CDK4/6 inhibitors, The MonarchE trial [70], enrolling 5637 patients with ER+/HER2–negative early-stage BC at high risk of recurrence according to tumor size, grade, ki67 expression and N status showed that adjuvant Abemaciclib for 2 years plus ET vs. ET alone improved IDFS (invasive disease-free survival) (HR 0.75 95% CI 0.60–0.93 *p* = 0.01) and DRFS (disease recurrence-free survival) (HR 0.72 95% CI 0.56–0.92 *p* = 0.01). Of interest, after the study treatment, per protocol ET maybe have been continued for 5 or 10 years, but no data on the outcome of these two subgroups have been reported so far.

The PALLAS trial [71] investigated the role of Palbociclib for 2 years plus ET vs. ET alone in the ER+/HER2-negative early-stage BC, but no difference was observed in IDFS between the two arms (*p* = 0.51).

The NataLEE trial [72] will evaluate Ribociclib for 3 years plus ET vs. ET alone in an adjuvant setting and is currently ongoing.

The role of PARP inhibitors in BC adjuvant therapy has been investigated by the OlympiA trial [73], a phase 3 randomized study that evaluated the role of poly(adenosine diphosphate-ribose) polymerase inhibitor Olaparib in HER2 negative early BC with BRCA1/BRCA2 germline pathogenic or likely pathogenic variant at high risk of recurrence. A total of 1836 patients, of which 18% were ER+, were randomized to receive olaparib vs. placebo in the adjuvant setting after neoadjuvant/adjuvant chemotherapy. Olaparib improved IDFS (HR 0.58 99.5% CI 0.41–0.82 *p* < 0.001) and 3 years DDFS (distant disease-free survival) (HR 0.57 99.5% CI 0.39–0.83 *p* < 0.001). The efficacy of Olaparib in ER+ BC was confirmed in the subgroup analyses. In this study, ET was allowed in HR+ BC patients, but the duration of ET beyond 5 years was not specified. 

Further studies are needed to better understand the role of extending ET in patients receiving these novel adjuvant treatments.

## 4. Conclusions

The decision of whether or not to propose an extended treatment approach is not an easy task. Based on the evidence available, the decision should be individualized, based on the patient’s decision after careful discussion of the benefits and drawbacks of this approach with the patient. Parameters that could be taken into account are primary tumor size, axillary nodes, patient’s age, estrogen and progesterone receptor status, genomic risk of recurrence, and intrinsic subtype, the latter two when available. The CTS5 is also a useful biomarker that resumes some of the main clinicopathological parameters associated with extended therapy benefits. Genomic tests that might provide the most pertinent prognostic and/or predictive information are EndoPredict, BCI, and, to some degree, Prosigna, especially when intrinsic subtypes are provided (outside the USA). Finally, previous tolerability to the first 5 years of standard ET and bone health should always be taken into account. In any case, dedicated studies are needed to better select patients for this therapeutic strategy, so to minimize the risk of unnecessary toxicities and maximize adherence to treatment.

## Figures and Tables

**Table 1:** Main extended ET trials’ features and results.

Trial	Treatment Arms	Patient Number	Population	Follow-Up (Years)	DFS Absolute Benefit	HR	OS Absolute Benefit	HR
aTTom 2013	Tam for 5 y	6953	ER+/Any N	9	3%	ND	1.60%	ND
	Tam for 10 y							
ATLAS 2013	Tam for 5 y	12,894	ER+/Any N	8	3.70%	ND	2.80%	ND
	Tam for 10 y							
DATA 2022	Tam 2–3 y → Ana for 3 y	1912	ER+/Any N	10.1	3%	0.86	-	0.93
	Tam 2–3 y → Ana for 6 y							
MA17 2012	Tam for 5 y → PLB for 5 y	5187	ER+/Any N	5.4	4.60%	0.58	-	0.77
	Tam for 5 y → Letro for 5 y							
IDEAL 2018	Tam or AI or Tam/AI for 5 y → Letro for 2.5 y	1824	ER+/Any N	6.6	3.20%	0.96	0.50%	1.05
	Tam or AI or Tam/AI for 5 y → Letro for 5 y							
NSABP-B42 2020	AI or Tam/AI for 5 y → PLB	3966	ER+/Any N	9.3	4%	0.74	0.60%	0.97
	AI or Tam/AI for 5 y → Letro for 5 y							
MA17R 2016	AI or Tam/AI for 5 y → PLB	1918	ER+/Any N	6.3	4%	0.66	−1%	0.97
	AI or Tam/AI for 5 y → Letro for 5 y							
SOLE 2021	ET x 4/6 y → AI for 5 y (cont.)	4884	ER+/N+	7	0.10%	1.03	−1%	0.89
	ET x 4/6 y → AI for 5 y (int.)							
GIM4 2021	Tam x 2/3 y → Letro for 2/3 y	2056	ER+/Any N	11.7	5%	0.78	4%	0.77
	Tam x 2/3 y → Letro for 5 y							
ABCSG-16 2021	Tam or AI or Tam/AI x 4/6 y → Ana for 2 y	3484	ER+/Any N	8.8	0.30%	0.99	−0.20%	1.02
	Tam or AI or Tam/AI for 4/6 y → Ana for 5 y							

Tam, tamoxifen; AI, aromatase inhibitor; Ana, anastrozole; Letro, letrozole; PLB, placebo; N, axillary lymph nodes; y, years; ER, endocrine receptor; +, positive; cont., continuous; int., intermittent; ND, not determined; HR, hazard ratio; ET, endocrine therapy.

## Data Availability

No new data were created in this study.
